# Asymmetric Activation of the Primary Motor Cortex during Observation of a Mirror Reflection of a Hand

**DOI:** 10.1371/journal.pone.0028226

**Published:** 2011-11-29

**Authors:** Wataru Tominaga, Jun Matsubayashi, Makiko Furuya, Masao Matsuhashi, Tatsuya Mima, Hidenao Fukuyama, Akira Mitani

**Affiliations:** 1 Laboratory of Neurorehabilitation, Department of Human Health Sciences, Graduate School of Medicine, Kyoto University, Kyoto, Japan; 2 Human Brain Research Center, Graduate School of Medicine, Kyoto University, Kyoto, Japan; University of Florida, United States of America

## Abstract

Mirror therapy is an effective technique for pain relief and motor function recovery. It has been demonstrated that magnetic 20-Hz activity is induced in the primary motor cortex (M1) after median nerve stimulation and that the amount of the stimulus-induced 20-Hz activity is decreased when the M1 is activated. In the present study, we investigated how the image or the mirror reflection of a hand holding a pencil modulates the stimulus-induced 20-Hz activity in the M1. Neuromagnetic brain activity was recorded from 13 healthy right-handed subjects while they were either viewing directly their hand holding a pencil or viewing a mirror reflection of their hand holding a pencil. The 20-Hz activity in the left or the right M1 was examined after the right or the left median nerve stimulation, respectively, and the suppression of the stimulus-induced 20-Hz in the M1 by viewing directly one hand holding a pencil or by viewing the mirror image of the hand holding a pencil was assumed to indicate the activation of the M1. The results indicated that the M1 innervating the dominant hand was suppressed either by viewing directly the dominant hand holding a pencil or by viewing the mirror image of the non-dominant hand holding a pencil. On the other hand, the M1 innervating the non-dominant hand was activated by viewing the mirror image of the dominant hand holding a pencil, but was not activated by viewing directly the non-dominant hand holding a pencil. The M1 innervating either the dominant or the non-dominant hand, however, was not activated by viewing the hand on the side ipsilateral to the M1 examined or the mirror image of the hand on the side contralateral to the M1 exaimined. Such activation of the M1 might induce some therapeutic effects of mirror therapy.

## Introduction

Mirror therapy was first introduced to treat “phantom limb” pain in upper extremity amputees [1]. A mirror was used so that patients could see the mirror reflection of their intact hand superimposed on the phantom, and most of the patients felt moving sensation of the phantom limb while watching the mirror reflection of their intact hand movements. This technique was applied in rehabilitation of motor functions after stroke [2]. Patients performed bilateral movements while watching the mirror reflection of their intact hand movements and showed significant motor functional improvements of the affected hand. Recently, the efficacy of mirror therapy for patients with pain and/or motor dysfunction was confirmed in randomized control trial studies [3]–[5] as well as in clinical intervention studies [6]–[8]. These studies have indicated that mirror therapy is one of promising rehabilitation therapeutic interventions.

It has also been attempted to elucidate the neural mechanisms underlying mirror therapy and demonstrated that the mirror reflection has strong effects on our sensory and motor systems: Neuropsychological studies, using mirror-induced conflicts between vision and proprioception, have reported that the visual information through a mirror dominates over the proprioceptive information [9], [10]. Neurophysiological studies, using transcranial magnetic stimulation [11] and a lateralized readiness potential [12], have also shown that motor cortex is activated during observation of a mirror reflection.

Magnetoencephalographic (MEG) studies have reported that 20-Hz rhythmic activity is induced in the primary motor cortex (M1) after median nerve (MN) stimulation. The stimulus-induced 20-Hz activity decreases after the electrical stimulation, and subsequently exhibits an increase above the pre-stimulus baseline, and then gradually returns to the baseline [13]. The generator source of the stimulus-induced 20-Hz activity is estimated to be in the M1 [13], [14]. The 20-Hz activity is abolished when the subjects are executing movements [13], [15], and is partially suppressed by various tasks related to movement such as action observation [16]–[18] and motor imagery [14]. These findings indicate that the suppression of 20-Hz activity represents the activation of the M1, and several studies have used it as an indicator of the functional state of the motor cortex [17], [18].

In our previous study, we examined whether the stimulus-induced 20-Hz activity of the left M1 might be suppressed when a subject viewed directly their hand holding a pencil or viewed the mirror image of their hand holding a pencil [19]. The results showed that the 20-Hz activity in the left M1 (the M1 contralateral to the dominant hand) was suppressed by viewing directly the right hand (dominant hand) holding a pencil as well as by viewing the mirror image of the left hand holding a pencil. In our previous study, however, the stimulus-induced 20-Hz activity of the right M1 (the M1 ipsilateral to the dominant hand) was not examined, and it still remains to be settled whether or not the stimulus-induced 20-Hz activity in the M1 ipsilateral to the dominant hand might be suppressed by viewing the right hand holding a pencil or by viewing the mirror image of the left hand holding a pencil. Thus, in the present study, we attempted to examine the effects of viewing one hand holding a pencil or the mirror image of the hand holding a pencil upon the 20-Hz activity in the M1 on both sides and to elucidate a potential functional mechanism mediating the effects of mirror therapy.

## Methods

### Ethics Statement

The study was performed in conformity with the Declaration of Helsinki, and approved by the Ethics Committee of the Kyoto University Graduate School and Faculty of Medicine. All subjects gave written informed consent prior to participation.

### Subjects

The experiments were carried out on 13 healthy subjects (nine females and four males, age range: 19–34, mean ± SD = 26±5) whose maximal temporal spectral evolution (TSE, see the section on the data analysis) levels of the stimulus-induced 20-Hz activity under the rest condition were above 120% of the baseline levels in both hemispheres. All subjects were right-handed, as confirmed by the Edinburgh Handedness Inventory [20] with a laterality score of 96±9 (mean ± SD, range: 70–100).

### Experiment paradigm

**Figure 1:**
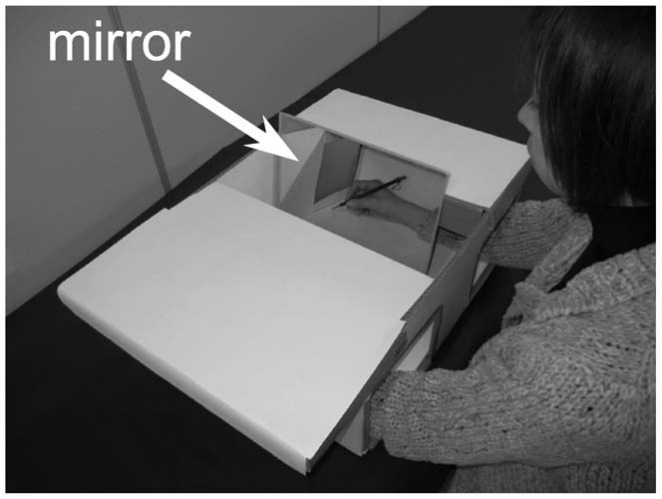
The mirror box used for producing mirror image of a hand holding a pencil. The subject inserted both hands into the box and viewed, through an opening in the top of the box, directly their hand holding a pencil or the mirror reflection of their hand holding a pencil. The unnecessary views of hands were prevented by using sliding boards on the top of the box. In this figure, a subject views the mirror image of her left hand. When the subject viewed directly their hand, transparent plastic was put in place of the mirror.

Subjects were seated in a chair in a magnetically shielded room, with both hands inserted into a mirror box in front of them. The design of the mirror box was the same as described in our previous study [19]. In brief, the mirror box was constructed by attaching a 25 cm by 30 cm mirror inside at an angle of 15–20° lateral to the sagittal plane. The position of the angle of the mirror was carefully adjusted so that the left or right hand looked like the right or left hand, respectively (Figure 1). The subjects viewed their hand holding a pencil or the mirror image of their hand holding a pencil through an opening in the top of the box.

**Figure 2:**
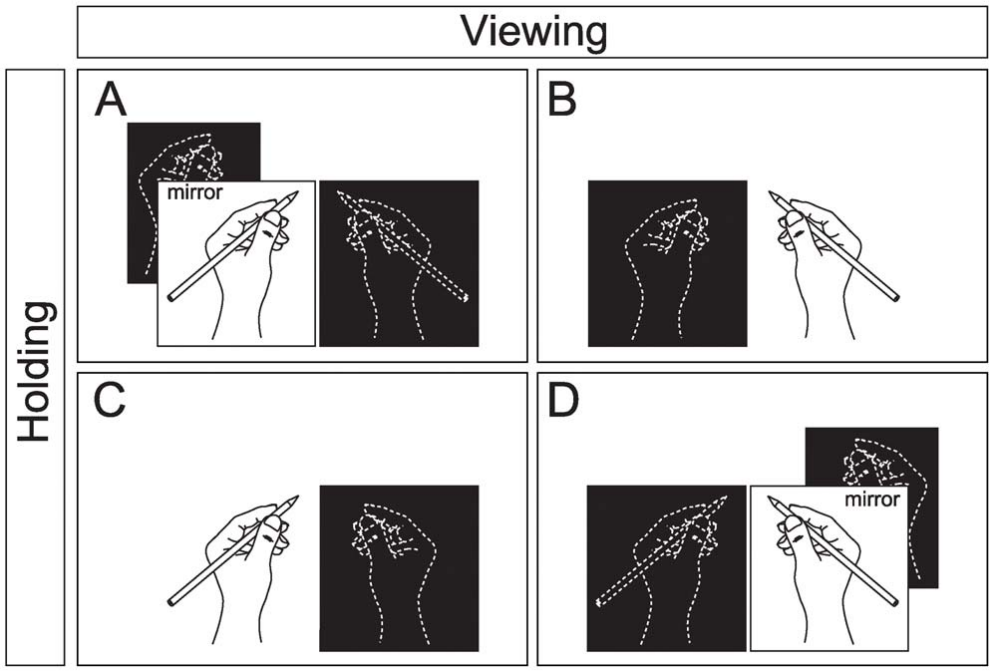
Schematic illustration of the experimental conditions. (A) viewing the mirror image of right hand: The subjects viewed the mirror image of their right hand holding a pencil. (B) viewing the right hand: The subjects viewed their right hand holding a pencil through transparent plastic. (C) viewing the left hand: The subjects viewed their left hand holding a pencil through transparent plastic. (D) viewing the mirror image of left hand: The subjects viewed the mirror image of their left hand holding a pencil. The hands covered with sliding boards are shown by white dashed lines on black background.

The experiment consisted of a rest condition and four experimental conditions (Figure 2). The subjects held a pencil softly so as not to produce any distinct muscle activity under the experimental conditions.

rest: The subjects rested while holding nothing and looked at a fixation point on the bottom of the box.

viewing the right hand: The subjects viewed their right hand holding a pencil through transparent plastic (25 cm×30 cm).

viewing the mirror image of right hand: The subjects viewed the mirror image of their right hand holding a pencil as their left hand holding a pencil.

viewing the left hand: The subjects viewed their left hand holding a pencil through transparent plastic.

viewing the mirror image of left hand: The subjects viewed the mirror image of their left hand holding a pencil as their right hand holding a pencil.

The right and left MNs were separately stimulated during the MEG recording to produce the stimulus-induced 20-Hz activity. Electrical stimulation was applied through a bipolar electrode placed on the right and left wrists. The stimuli were 0.3-ms constant-current pulses once every 1.5 s and the intensity was 120% of the motor threshold in each subject (right wrist: 2.2–5.8 mA, mean ± SD = 3.8±0.8 mA; left wrist: 2.1–6.1 mA, mean ± SD = 4.1±1.1 mA).

The MEG recording was carried out during eight sessions. Right or left MN stimulation was delivered. In each session, subjects performed one of the four experimental conditions. Each experimental condition was performed twice. An experimental period of 15 s was continually repeated four times with short breaks during each session. The subjects were instructed to concentrate on viewing their hand holding a pencil or the mirror image of their hand holding a pencil during each experimental period. The beginning and end of each experimental period were notified through an intercom. The subjects put down the pencil after the end of the fourth experimental period and looked at the fixation point during the resting period (rest condition). After that, the side of MN stimulation was switched and the same sequence of measurement was repeated. Each session took about 4 min. Then, the next experimental condition was performed after a short intervening pause. The order of the experimental conditions was balanced across subjects.

### Recording

Cortical magnetic signals were recorded with a 306-channel whole-head neuromagnetometer (Vectorview; Elekta Neuromag, Finland), which has 204 planar gradiometers and 102 magnetometers. In this study, the signals measured with the gradiometers were used for analysis. The recording passband was 0.03–330 Hz and the signals were digitized at 1003 Hz and stored for off-line analysis.

Four head-position-indicator coils were attached on the scalp to determine the head position of the subjects with respect to the sensor array of the neuromagnetometer. The locations of the coils and landmarks (the two preauricular points and the nasion) were digitized using a 3-D digitizer (Isotrak 3S1002, Polhemus Navigation Sciences, Colchester, VT) prior to the MEG recording. The subjects were instructed not to move their head during the MEG recording and the changes in head position during the recording were allowed to be within 5 mm in each axis.

Surface electromyograms (EMGs) were recorded to check the relaxation of the subject's hands. Pairs of cup electrodes were placed over the extensor digitorum communis muscles of both hands. Inter-electrode distance was approximately 3 cm. The EMGs were continuously monitored during the MEG recording, and the subjects were instructed to relax the hands when any distinct muscle activity was observed. Vertical electrooculogram and the markers indicating the delivery of the stimuli were also recorded.

### Data analysis

The modulation of the 20-Hz activity was quantified by the temporal spectral evolution (TSE) method [13]. The raw signals were visually inspected to reject epochs containing external noise, blinks, eye movements, or excessive muscle contraction. The signals were filtered through 18–23 Hz, and then rectified. These signals were averaged with respect to the onset of stimulus, from 0.2 s before the onset of the stimulus to 1.3 s after the onset, and the root-mean-square value of the signals from two gradiometers measuring orthogonal derivatives of the magnetic field at each location was calculated, and finally smoothed with a 15-Hz low-pass filter. Because a planar gradiometer shows the strongest signal where the gradiometer is just above a tangential cortical source [21], the TSE signals from the most reactive sensor pair under the rest condition were used for the following analysis. The mean TSE amplitudes in a time window from −0.2 to −0.1 s and in a time window from 0.3 to 0.8 s were used as the baseline level and the TSE level of the 20-Hz activity under each condition, respectively. The ratios of the TSE level under each of the four experimental conditions to that under the rest condition were also calculated and expressed in percentage form in each subject [13].

The mean values of the ratios of the TSE level under the four experimental conditions were compared with a two-way repeated measures analysis of variance (ANOVA) using “Holding” (Which hand is holding a pencil?) and “Viewing” (Which hand looks like it is holding a pencil?) as factors in each hemisphere. In the case that interaction between Viewing and Holding was significant, simple main effects were analyzed using a one-way repeated measures ANOVA. Significance was set at p<0.05.

## Results

### 20-Hz activity under the rest condition

The bursts of 20-Hz activity were induced by MN stimulation. About 70–75 epochs under each condition were averaged with respect to the onset of stimulus.

**Figure 3:**
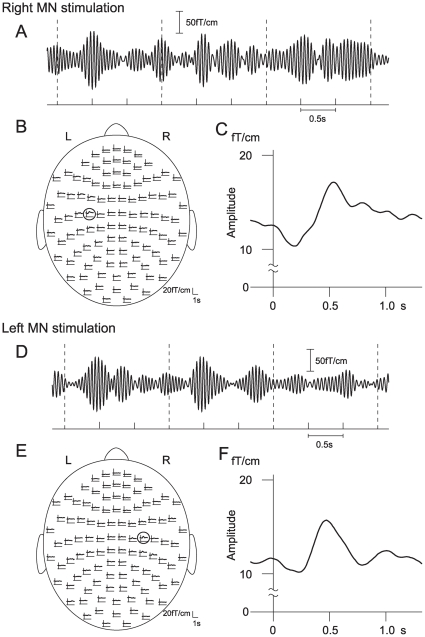
MEG activity induced by MN stimulation in a subject under the rest condition. Bursts of 20-Hz activity were induced after right MN stimulation (A–C) and left MN stimulation (D–F). A, D: Signals were recorded from the channel over the left M1 after right MN stimulation (A) and over the right M1 after left MN stimulation (D). The signals were bandpass-filtered through 18–23 Hz. Vertical dashed lines indicate MN stimuli delivered once every 1.5 s. Note that the stimulus-induced 20-Hz activity is enhanced after a short suppression period. B, E: The 20-Hz activity was quantified by TSE method. TSE curves show the distribution of the 20-Hz activity after right MN stimulation (B) and after left MN stimulation (E). The curves show the time course of changes in the root-mean-square values of TSE signals from the gradiometer pair at each location from 0.2 s before the onset of the stimuli to 1.3 s after the onset. Vertical lines indicate the onset of the MN stimuli. The heads are viewed from the top. Note that prominent 20-Hz activity was induced in the left M1 corresponding to the hand area (encircled in B) and the right M1 corresponding to the hand area (encircled in E). C, F: The TSE curves obtained from the most reactive gradiometer pair encircled in B (C) and encircled in E (F). Ordinates, 20-Hz activity levels (fT/cm); abscissas, time before and after the onset of MN stimulation.

The 20-Hz activity was slightly suppressed immediately after MN stimulation and then the activity started to be enhanced (Figure 3A, D). TSE curves showed the distribution of the enhancement of 20-Hz activity after right and left MN stimulation under the rest condition (Figure 3B, E). The enhancement of the stimulus-induced 20-Hz activity was most prominent in the region corresponding to the hand area of the M1 contralateral to the MN stimulation (encircled in Figure 3B, E). The enhancement of the stimulus-induced 20-Hz activity was larger in the left M1 than in the right M1 in each subject. The amount of 20-Hz activity peaked around 0.5 s after MN stimulation and slowly decayed until the next stimulus (Figure 3C, F). The distribution and time course of the stimulus-induced 20-Hz activity were similar to those which have been reported in the previous studies [13], [14], [18], [19].

### 20-Hz activity under the experimental conditions

**Figure 4:**
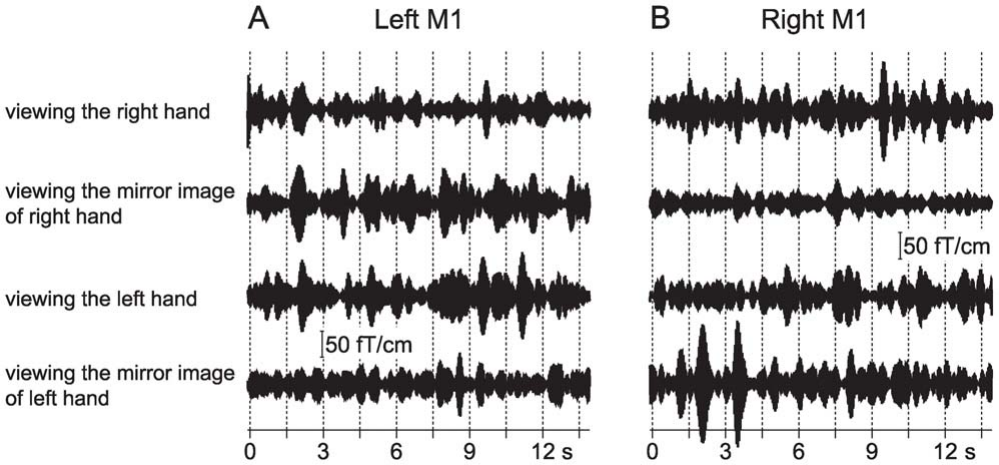
20-Hz activity in the M1 of a subject under each of the four experimental conditions. The MEG signals were recorded over the left M1 after right MN stimulation (A) and the right M1 after left MN stimulation (B) under experimental conditions (viewing the right hand, viewing the mirror image of right hand, viewing the left hand, and viewing the mirror image of left hand). The signals were bandpass-filtered through 18–23 Hz. Vertical dashed lines indicate MN stimuli delivered once every 1.5 s.

The stimulus-induced 20-Hz activity was variable according to the experimental conditions (Figure 4). In the left M1 (Figure 4A), the 20-Hz activity induced by right MN stimulation (Figure 4A) was suppressed when the subjects viewed directly their right hand holding a pencil or viewed the mirror image of their left hand holding a pencil. The 20-Hz activity, however, was not suppressed when the subjects viewed directly their left hand holding a pencil or viewed the mirror image of their right hand holding a pencil. On the other hand, the 20-Hz activity which was induced in the right M1 by left MN stimulation (Figure 4B) was strongly suppressed when the subjects viewed the mirror image of their right hand holding a pencil but was not significantly suppressed when the subjects viewed directly their right hand holding a pencil. It was also revealed that the 20-Hz activity induced in the right M1 was not suppressed by viewing directly the left hand holding a pencil or by viewing the mirror image of the left hand holding a pencil.

**Figure 5:**
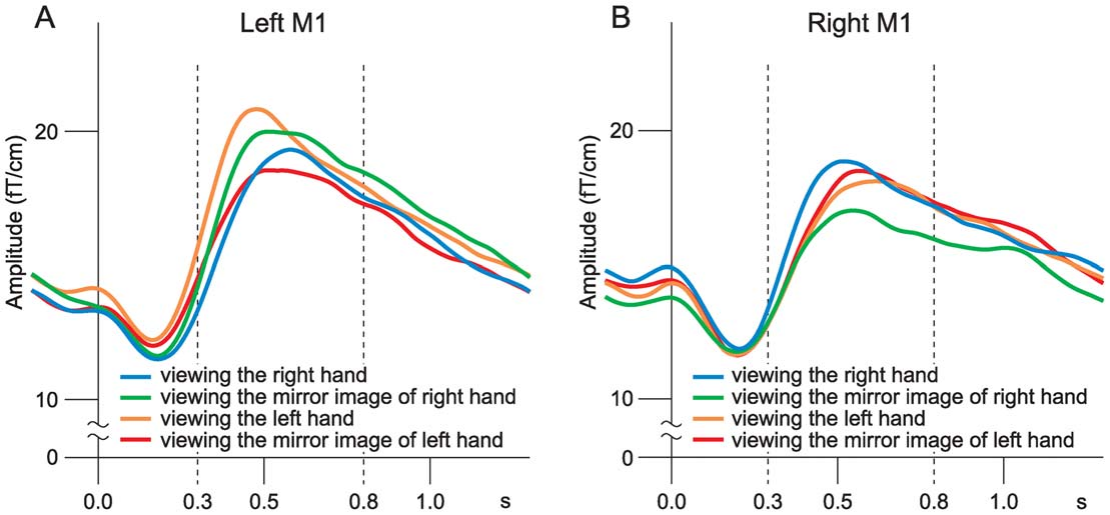
TSE curves obtained across 13 subjectes under each of the four experimental conditions. The TSE curves were obtained across 13 subjects in the left M1 (A) and the right M1 (B). Blue, green, orange, and red lines indicate TSE curves under the four experimental conditions: viewing the right hand, viewing the mirror image of right hand, viewing the left hand, and viewing the mirror image of left hand, respectively. The vertical axis shows TSE signal amplitude (fT/cm); the horizontal axis shows time before and after the onset of MN stimulation.

The 20-Hz activity was quantified by the TSE method: The mean values of the TSE levels in the left M1 were estimated in a time window from 0.3 to 0.8 s after MN stimulation across the 13 subjects under each of the four experimental conditions (Figure 5A). When the subjects viewed directly their right hand holding a pencil, the mean value of the TSE levels was 17.8±2.7 fT/cm (± SEM) (blue line in Figure 5A) and the mean value of the ratios obtained from each subject was 94.3±3.7% (± SEM). When the subjects viewed the mirror image of their left hand holding a pencil, the mean value of the TSE levels was 17.7±2.4 fT/cm (red line in Figure 5A) and the mean value of the ratios was 95.0±1.7%. On the other hand, when the subjects viewed directly their left hand holding a pencil, the mean values of the TSE levels was 19.2±2.9 fT/cm (orange line in Figure 5A) and the mean value of the ratios was 101.9±1.2%. When the subjects viewed the mirror image of their right hand holding a pencil, the mean value of the TSE levels was 18.8±2.6 fT/cm (green line in Figure 5A), and the mean value of the ratios was 101.2±2.5%. The mean values of the ratio of the TSE levels under the four experimental conditions were compared with a two-way repeated measures ANOVA (Viewing x Holding); a significant main effect was revealed in Viewing (F(1,12) = 10.093, p = 0.008), but not in Holding (F(1,12) = 0.106, p = 0.751). It was further revealed that the interaction between Viewing and Holding was not significant (F(1,12)<0.001, p = 0.986), and indicated that the difference on the effect of Viewing was not affected by the effect of Holding. These results indicated that, the 20-Hz activity induced in the M1 innervating the dominant hand was suppressed when the subjects viewed directly their dominant hand holding a pencil or viewed the mirror image of their non-dominant hand holding a pencil looking like their dominant hand holding a pencil, but it was not suppressed when the subjects viewed directly their non-dominant hand holding a pencil or viewed the mirror image of the their dominant hand holding a pencil looking like their non-dominant hand holding a pencil.

As regards the right M1, the mean values of the TSE levels were estimated in the same way as in the left M1 (Figure 5B): When the subjects viewed the mirror image of their right hand holding a pencil, the mean value of the TSE levels was 16.1±1.9 fT/cm (green line in Figure 5B) and the mean value of the ratios was 89.5±2.1%. In contrast, when the subjects viewed directly their right hand holding a pencil, the mean value of the TSE levels was 17.6±1.9 fT/cm (blue line in Figure 5B) and the mean value of the ratios was 99.3±1.6%. When the subjects viewed directly their left hand holding a pencil, the mean value of the TSE levels was 16.9±1.7 fT/cm (orange line in Figure 5B) and the mean value of the ratios was 96.1±1.7%. When the subjects viewed the mirror image of their left hand holding a pencil, the mean value of the TSE levels was 17.2±1.7 fT/cm (red line in Figure 5B) and the mean value of the ratios was 98.6±2.9%. A two-way repeated measures ANOVA (Viewing x Holding) revealed a significant main effect in Viewing (F(1,12) = 8.412, p = 0.013), but not in Holding (F(1,12) = 1.740, p = 0.212). On the other hand, the interaction effect between Viewing and Holding was significant (F(1,12) = 5.227, p = 0.041), indicating that the difference on the effect of Viewing was affected by the effect of Holding. Thus, simple main effects were further analyzed with a one-way repeated measures ANOVA, setting the significance at p<0.05. The analysis revealed that the TSE level observed during viewing the mirror image of right hand was significantly lower than that observed during viewing the left hand (F(1,12) = 5.324, p = 0.040), the right hand (F(1,12) = 15.814, p = 0.002), or the mirror image of left hand (F(1,12) = 5.865, p = 0.032). The comparisons among three other experimental conditions (viewing the left hand, viewing the mirror image of left hand, or viewing the right hand) did not show any significance (viewing the left hand vs. viewing the mirror image of left hand: F(1,12) = 0.800, p = 0.389; viewing the mirror image of left hand vs. viewing the right hand: F(1,12) = 0.076, p = 0.787; viewing the right hand vs. viewing the left hand: F(1,12) = 2.227, p = 0.161). These results indicated that the 20-Hz activity induced in the right M1 after left MN stimulation was suppressed by viewing the mirror image of the right hand holding a pencil, and that it was not significantly suppressed by viewing directly the right or left hand holding a pencil or by viewing the mirror image of left hand holding a pencil.

## Discussion

It has been reported that the stimulus-induced 20-Hz activity is almost completely suppressed during the execution of hand movements [13], [15] and partially suppressed during its motor imagery which is defined as conscious mental rehearsal of a motor act without performing any overt movement [14]. These reports indicate that the M1 is strongly activated during the execution of actual movements and partially activated during mental rehearsal of a motor act. The M1 has also been reported to be partially activated during observation of another person's hand movements [16]–[18] and of a mirror reflection of a hand [11], [12]. Järveläinen et al. have shown that the stimulus-induced 20-Hz activity was strongly suppressed when the subject observed another person placing small objects with chopsticks from one dish to another whereas it was weakly suppressed when the subjects observed another person doing similar movements without touching or moving the objects [17]. Moreover, Ichikawa et al. have shown that the stimulus-induced 20-Hz activity was strongly suppressed when the subjects observed another person's hand movements presented in the same direction as the subject's hand whereas it was weakly suppressed when the subject observed similar hand movements presented in the opposite direction to the subject's hand [18]. These reports suggest that the stimulus-induced 20-Hz activity is strongly suppressed during observation of movements closely related to the subject's own movements. The subjects may unconsciously perform a mental rehearsal of a motor act during observation of the movements closely related to the subject's own movements.

In the present study, neuromagnetic brain activity was recorded from 13 healthy right-handed subjects while they were either viewing directly their hand holding a pencil or viewing a mirror image of their hand holding a pencil. The 20-Hz activity in the left or the right M1 was examined after the right or the left median nerve stimulation, respectively. Confirming the results of our previous study [19], the present study showed that the stimulus-induced 20-Hz activity in the left M1 was suppressed when the subjects viewed directly the right hand holding a pencil or viewed the mirror image of the left hand holding a pencil, so that it appeared to be the right hand holding a pencil. The present study further demonstrated that the stimulus-induced 20-Hz activity in the right M1 was suppressed when the subjects viewed the mirror image of their right hand looking like the left hand holding a pencil, but was not significantly suppressed when the subjects viewed directly their left hand holding a pencil. The stimulus-induced 20-Hz activity was not suppressed in both the right and left M1 when the subjects viewed the hand holding a pencil on the side ipsilateral to the M1 examined or when they viewed the mirror image of the hand holding a pencil looking like the ipsilateral hand holding a pencil.

Thus, the present study indicated that the M1 innervating the dominant hand was activated either by viewing directly the dominant hand holding a pencil or by viewing the mirror image of the non-dominant hand holding a pencil looking like the dominant hand holding a pencil. On the other hand, the M1 innervating the non-dominant hand was activated by viewing the mirror image of the dominant hand holding a pencil, but was not activated by viewing directly the non-dominant hand holding a pencil. The M1 innervating the non-dominant hand appeared to be activated more strongly by viewing the mirror image of the dominant hand holding a pencil than by viewing directly the non-dominant hand holding a pencil. The M1 innervating either the dominant or the non-dominant hand, however, was not activated by viewing the hand on the side ipsilateral to the M1 examined or the mirror image of the hand on the side contralateral to the M1 examined.

The dominant hand has a high function for writing; therefore, the mental rehearsal of writing might be unconsciously induced in the M1 innervating the dominant hand by viewing directly the dominant hand holding a pencil or by viewing the mirror image of the non-dominant hand holding a pencil looking like the dominant hand holding a pencil. In the M1 innervating the non-dominant hand, the mental rehearsal of writing might be more strongly induced by viewing the mirror image of the dominant hand holding a pencil than by viewing directly the non-dominant hand holding a pencil.

In summary, the M1 innervating the dominant hand is activated by viewing directly the dominant hand holding a pencil or viewing the mirror image of the non-dominant hand holding a pencil. The M1 innervating the non-dominant hand is also activated by viewing the mirror image of the dominant hand holding a pencil. Some therapeutic effects of mirror therapy might be ascribable, at least partly, to such activation of the M1 innervating the dominant or the non-dominant hand.
